# Induction and inhibition of oocyte maturation by EDCs in zebrafish 

**DOI:** 10.1186/1477-7827-3-69

**Published:** 2005-12-09

**Authors:** Toshinobu Tokumoto, Mika Tokumoto, Yoshitaka Nagahama

**Affiliations:** 1 Department of Biology and Geosciences, Faculty of Science, National University Corporation Shizuoka University, Shizuoka 422-8529, Japan; 2 CREST Research Project, Japan Science and Technology Corporation, Kawaguchi 332-0012, Japan; 3 Laboratory of Reproductive Biology, National Institute for Basic Biology, Okazaki 444-8585, Japan

## Abstract

**Background:**

Oocyte maturation in lower vertebrates is triggered by maturation-inducing hormone (MIH), which acts on unidentified receptors on the oocyte surface and induces the activation of maturation-promoting factor (MPF) in the oocyte cytoplasm. We previously described the induction of oocyte maturation in fish by an endocrine-disrupting chemical (EDC), diethylstilbestrol (DES), a nonsteroidal estrogen.

**Methods:**

In this study, stimulatory and inhibitory effects of EDCs and natural steroids on oocyte maturation were examined in zebrafish. For effective agents, some details about the mechanism in induction or inhibition of maturation were examined. Possible groups of DES interacting with the MIH receptor are discussed based on relative potency of steroids to induce maturation.

**Results:**

Among agents tested, tamoxifen (TAM) and its metabolite 4-hydroxytamoxifen (4-OHT) showed stimulatory activity similar to DES. The time courses of the change in germinal vesicle breakdown and an intracellular molecular event (the synthesis of cyclin B) induced by TAM were indistinguishable from those induced by MIH. In contrast, pentachlorophenol (PCP) had a potent inhibitory effect on MIH-induced oocyte maturation. PCP inhibited not only MIH-induced maturation but also DES- and TAM-induced maturation. Methoxychlor also inhibited maturation when oocytes were pre-treated with this agent.

**Conclusion:**

These results suggest that EDCs act as agonists or antagonists in the induction of oocyte maturation in fish.

## Background

Fish oocytes provide an appropriate experimental system with which to investigate the molecular mechanisms controlling meiosis and the embryonic cell cycle. Several factors responsible for the regulation of meiotic maturation in fish oocytes have been identified. These include the isolation and characterization of a fish maturation-inducing hormone (MIH) and the components of maturation-promoting factor (MPF) (cdc2, the catalytic subunit, and cyclin B, the regulatory subunit) [1]

Oocyte maturation in fish is triggered by MIH, which acts on receptors located on the oocyte membrane and induces the activation of MPF in the oocyte cytoplasm [2]. During the course of maturation, oocytes undergo drastic morphological changes associated with progression of the meiotic cell cycle, among which breakdown of the oocyte nuclear envelope (germinal vesicle breakdown, GVBD) occurring at the prophase/metaphase transition is usually regarded as a hallmark of the progress of oocyte maturation. In a number of teleost species, C21 steroids have been shown to be potent initiators of GVBD *in vitro *and to be present at high levels in plasma of fish undergoing final oocyte maturation. Among C21 steroids, however, only two were identified as naturally occurring MIH in fish: 17α, 20β-dihydroxy-4-pregnen-3-one (17, 20β-DHP) in amago salmon [3] and 17α, 20β, 21-trihydroxy-4-pregnen-3-one (20β-S), in the Atlantic croaker and spotted sea trout [4]. Testosterone, as well as other C19 steroids, induces GVBD only at high concentrations. Estradiol-17β and other C18 steroids are generally not effective inducers of oocyte maturation in fish [5]. Recently, a strong candidate for the MIH receptor, membrane progestin receptor (mPR), was identified [6,7]. In zebrafish, two types of mPRs, α and β, were identified [8]. 17α, 20β-DHP has been shown to induce oocyte maturation by stimulating the *de novo *synthesis of cyclin B, a regulatory subunit of MPF [9].

Several endocrine-disrupting chemicals or EDCs, Kepon and o,p-DDD, have been reported to antagonize MIH-induced meiotic maturation of fish oocytes *in vitro *[10]. EDCs such as methoxychlor and ethynyl estradiol also antagonize frog oocyte maturation. One of EDCs, diethylstilbestrol (DES), is a nonsteroidal substance which was prescribed during the late 1940s to early 1970s to pregnant women to prevent abortion, preeclampsia, and other complications of pregnancy. Male and female offspring exposed in utero to DES may develop multiple dysplastic and neoplastic lesions of the reproductive tract, along with other changes, during development [11]. In a previous study, we found that treatment of oocytes with DES alone induces maturation in goldfish and zebrafish [12]. The results suggested that DES might interact with mPR to induce maturation. In the present study, we examined stimulatory and inhibitory effects of other EDCs on zebrafish oocyte maturation and discussed possible groups of DES interacting with the MIH receptor.

## Methods

### Materials

Zebrafish were purchased from aquatic dealer and were maintained at 28.5°C on a 14 h light/10 h dark cycle [13]. 17, 20β-DHP, DES, TAM, 4-OHT and 17β-estradiol were purchased from Sigma Chemical Co. (St. Louis, MO). 17α-Estradiol, ethynylestradiol, butyl benzyl phthalate, di (2-ethylhexyl) phthalate and pentachlorophenol were obtained from Wako Pure Chemical Industries (Osaka, Japan). Other chemicals were purchased as follows: resveratorol, Calbiochem (Darmstadt, Germany); DDTs, AccuStandard (New Haven, CT); bisphenol A, Nacalai Tesque (Kyoto, Japan); p-nonylphenol, Kanto Chemical Co. (Tokyo, Japan); 4-octylphenol, Aldrich Chemical Co. (Milwaukee, WI).

### Oocyte preparation and *in vitro *culture

Gravid female zebrafish which possesses full-grown immature oocytes were selected from a group of mixture of 10–50 male and female that were keep in 20 cm × 25 cm square and 25 cm high acryl case with continuous out-flow water. Ovaries of zebrafish were isolated from sacrificed females and placed in fresh zebrafish Ringer's solution (116 mM NaCl, 2.9 mM KCl, 1.8 mM CaCl_2_, and 5 mM HEPES, pH 7.2) and washed with the same solution. Ovaries were dissected into ovarian fragments (each containing 2–10 oocytes) manually by using fine forceps. Fully-grown immature oocytes were exposed *in vitro *by incubating ovarian fragments in 4 ml of zebrafish Ringer's solution containing each agent (from a 1000-fold stock in ethanol) at 25.0°C or room temperature with gentle agitation (40 rpm). To assess the maturation processes, germinal vesicles (GVs) were examined under a binocular microscope (SMZ645, Nikon, Tokyo, Japan) after placing the oocytes in clearing solution [14] or GVBD was assessed by scoring the oocytes that became transparent [15]. %GVBD was determined in more than twenty oocytes.

### Preparation of oocyte and egg extracts

Intact follicles were carefully isolated by using fine forceps. Groups of twenty intact follicles were transferred to a 1.5-ml Eppendorf micro centrifuge tube and crushed with 5 strokes of a plastic pestle in 200 μl of sample buffer for SDS-PAGE. The samples were centrifuged at 5,000 rpm for 5 min at 4°C in a fixed-angle rotor (MX-300 micro centrifuge, TOMY, Tokyo, Japan). The supernatant (100 μl) was collected for electrophoresis and immunoblotting.

### SDS-PAGE and immunoblotting

Antiserums, which recognize the protein band of zebrafish cyclin B1 and mPRα produced in previous study were used [12]. Proteins were separated by polyacrylamide gel electrophoresis under denaturing conditions (SDS-PAGE with 10% gel) by the method of Laemmli [16], and transferred to Immobilon membrane (Millipore, Billerica, MA). Membranes were blocked in 5% non-fat powdered milk, and incubated with primary antibodies for 1 hr at room temperature. Immunocomplexes were visualized using the ECL detection kit (Amersham Biosciences, Uppsala, Sweden).

### Statistical Analysis

**Figure 1:**
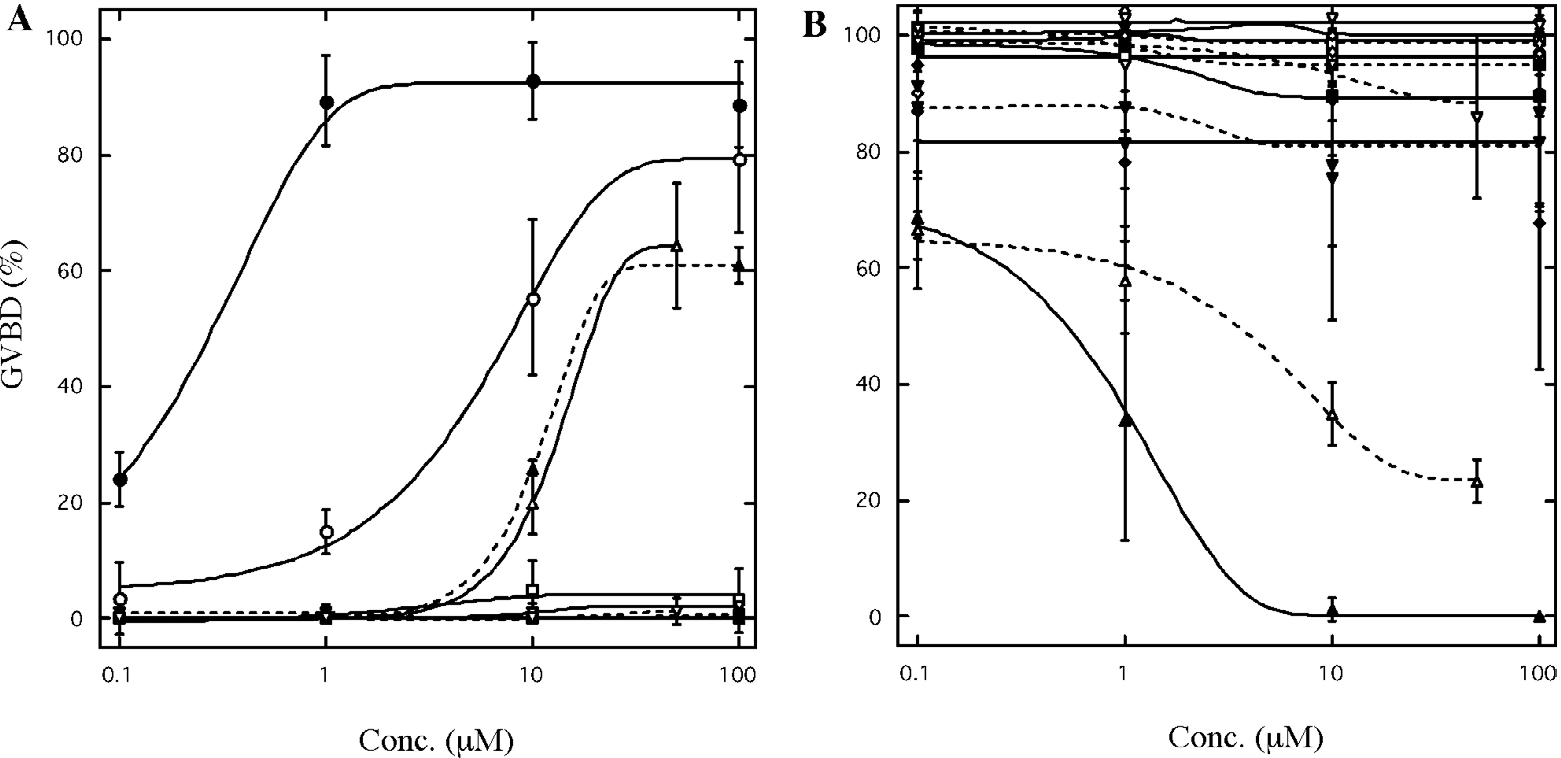
**Induction and inhibition of oocyte maturation by EDCs**. (A) Oocyte maturation-inducing activity of EDCs was examined. Each value is the mean of three separate experiments using ovaries of three different females except for TAM and 4-OHT (n = 6). (B) Effects of EDCs on 17,20β-DHP-induced oocyte maturation were examined. Each compound was added to the medium, then 0.1 μM of 17, 20β-DHP was applied. %GVBD was calculated by determining the percentage of oocytes that had undergone germinal vesicle breakdown (GVBD) among more than 20 oocytes cultured for three hours. Each value is the mean of three separate experiments using ovaries of three different females. Vertical lines indicate standard deviation. A logistic function was used to fit the dose-dependence. Each symbols represents as following: —●—, 17,20β-DHP; —○—, DES; --■--, 17α-Estradiol; —□—, 17β-estradiol; --■--, ethynylestradiol; --□--, resveratorol; —◆—, o,p'-DDT; --◆--, p,p'-DDT; —◇—, butyl benzyl phthalate ; ----◇--, di(2-ethylhexyl)phthalate; —▲—, pentachlorophenol; —▼—, bisphenol A; --▼--, p-nonylphenol; —▽—, octylphenol; --▲--,TAM; —△—, 4-OHT; --▽--, methoxychlor; --△-- methoxychlor (1 hr pre-incubation).

Summary data are presented as mean ± S.D. Student's t test was used to determine the statistical significance of the obtained data. The significance between multiple groups of the data in Figure 1 was evaluated using analysis of variance (ANOVA). Data were considered significant at P < 0.05(*) or P < 0.01(**).

## Results

### Relative potency of various substances in inducing and preventing fish oocyte maturation

The relative effectiveness of 17,20β-DHP and sixteen other agents including EDCs and several steroid hormones in inducing and preventing GVBD was investigated using zebrafish oocytes (Fig. 1). As described previously, DES exhibited strong activity to induce GVBD like 17,20β-DHP. As predicted given their structural similarity to DES, TAM and 4-OHT were also effective in inducing GVBD. TAM and 4-OHT induced 10–20% GVBD at 10 μM. They were relatively less potent than DES and induced 60–70% GVBD even at the highest concentration. As shown in the goldfish oocytes [12], two estrogens did not induce GVBD at the concentration tested. Nine kinds of EDCs: DDTs, phthalates and phenols, did not induce GVBD as in goldfish. The inhibitory effects of EDCs on 17,20β-DHP-induced oocyte maturation were also examined. Of the fourteen agents tested, only PCP was effective in inhibiting GVBD without pre-incubation (Fig. 1B). When oocytes were treated with 10 μM PCP in addition to 17,20β-DHP, maturation was completely prevented. Methoxychlor also inhibited GVBD when oocytes were pre-incubated with this agent.

### EDCs induce natural oocyte maturation

**Figure 2:**
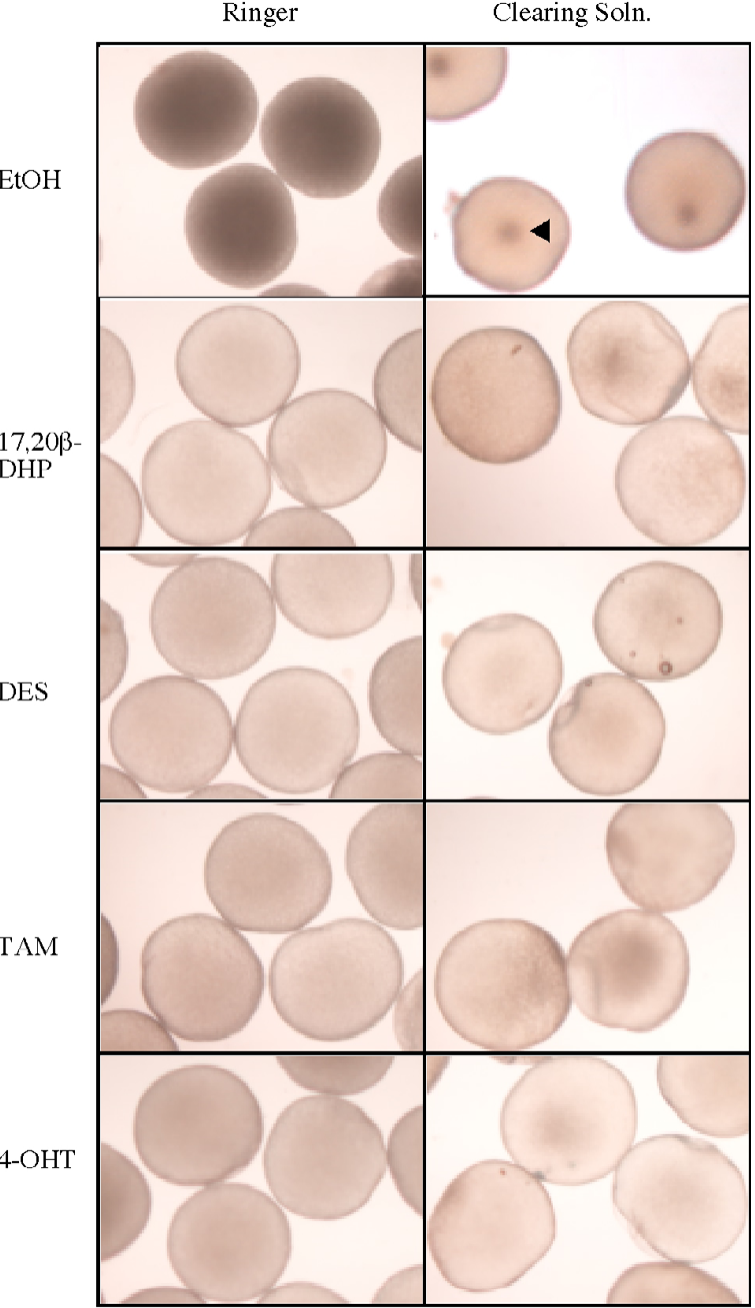
**EDCs induce zebrafish oocyte maturation**. The morphology of oocytes after three hours of each treatment was photographed. Left-side panels indicate oocytes in zebrafish Ringer's solution. In right-side panels, oocytes were fixed in clearing solution for observation of germinal vesicles. Oocytes remained opaque after EtOH treatment whereas they became transparent after 17α, 20β-DHP, DES, TAM or 4-OHT treatments. Germinal vesicles were seen near the center of oocytes after EtOH treatment whereas they disappeared after 17α, 20β-DHP, DES, TAM or 4-OHT treatments. The arrow indicates germinal vesicle.

**Figure 3:**
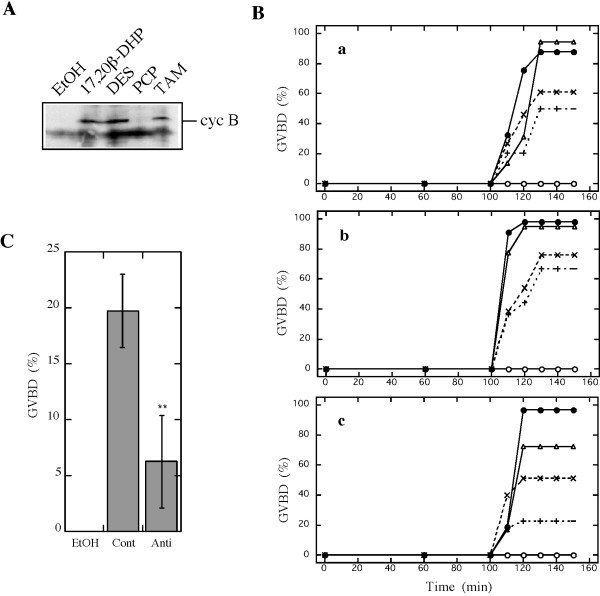
**Induction of maturation by tamoxifens**. (A) *De novo *synthesis of cyclin B. Extracts were prepared from twenty oocytes after incubation with EtOH, 17,20β-DHP, DES, TAM or PCP with 17,20β-DHP. Extracts of each treatment were electrophoresed under denaturing conditions (10.0% gel) and immunostained with anti-goldfish cyclin B polyclonal antibody after electroblotting. An arrow indicates the 48 kDa band of cyclin B. (B) Time-course change of germinal vesicle breakdown induced by EDCs. Oocyte maturation induced by 0.01% EtOH (○), 0.1 μM 17,20β-DHP (●), 1 μM DES (Δ) 100 μM TAM (×) or 50 μM 4-OHT (+). Each panel indicates separate experiments using three different females. (C) Inhibition of oocyte maturation by anti-mPRα antibody. Oocytes were incubated with 100 μg/ml of anti-goldfish mPRα polyclonal antibody (Anti) or control IgG (Cont) for 1 hour at 25°C, then oocytes were treated with 10 μM TAM for 3 hrs. %GVBD was calculated by determining the percentage of oocytes that had undergone germinal vesicle breakdown (GVBD) in a group of more than 20 oocytes. Each value is the mean of three separate experiments using ovaries from three separate females. Vertical bars show standard deviation. **indicates significant differences between the %GVBD induced in control- and anti-mPRα antibody-treated oocytes at the P < 0.01 level.

Figure 2 show the morphology of oocytes after three hours treatment with EtOH, 17,20β-DHP, DES, TAM and 4-OHT. Germinal vesicles were seen near the center of oocytes after EtOH treatment whereas they disappeared after the 17,20β-DHP and EDC treatments. Also, 17,20β-DHP- and EDC-treated oocytes became transparent. TAM also induced the translation of cyclin B protein (Fig. 3A), a well-characterized intracellular molecular event that results in an elevation of the kinase activity of MPF. These results show that the TAM-induced maturation and 4-OHT-induced maturation were identical to the physiological maturation of oocytes as described for DES-induced maturation. To identify the target of TAM and 4-OHT in the signal transduction pathway to induce GVBD, the time-course of changes in GVBD induced by TAM and 4-OHT was compared with that of GVBD induced by 17,20β-DHP and DES. The time course of oocyte maturation induced by TAM and 4-OHT was the same as that induced by 17,20β-DHP and DES (Fig. 3B), suggesting that the most likely candidate for the target of EDCs is a membrane progestin receptor of 17,20β-DHP. As previously described, 17,20β-DHP-induced and DES-induced oocyte maturation were both prevented by antibody against an N-terminal fragment of goldfish mPRα. Then, the effect of the antibody on the TAM-induced oocyte maturation was examined. Immature oocytes were treated with the antibody for goldfish mPRα before addition of TAM. The extent of GVBD was lowered to approximately 30% in the antibody-treated group, compared to the control IgG group (Fig. 3C). These results strongly suggested that TAM induces oocyte maturation via a MIH-mediated pathway the same as DES.

### Inhibition of oocyte maturation by PCP

**Figure 4:**
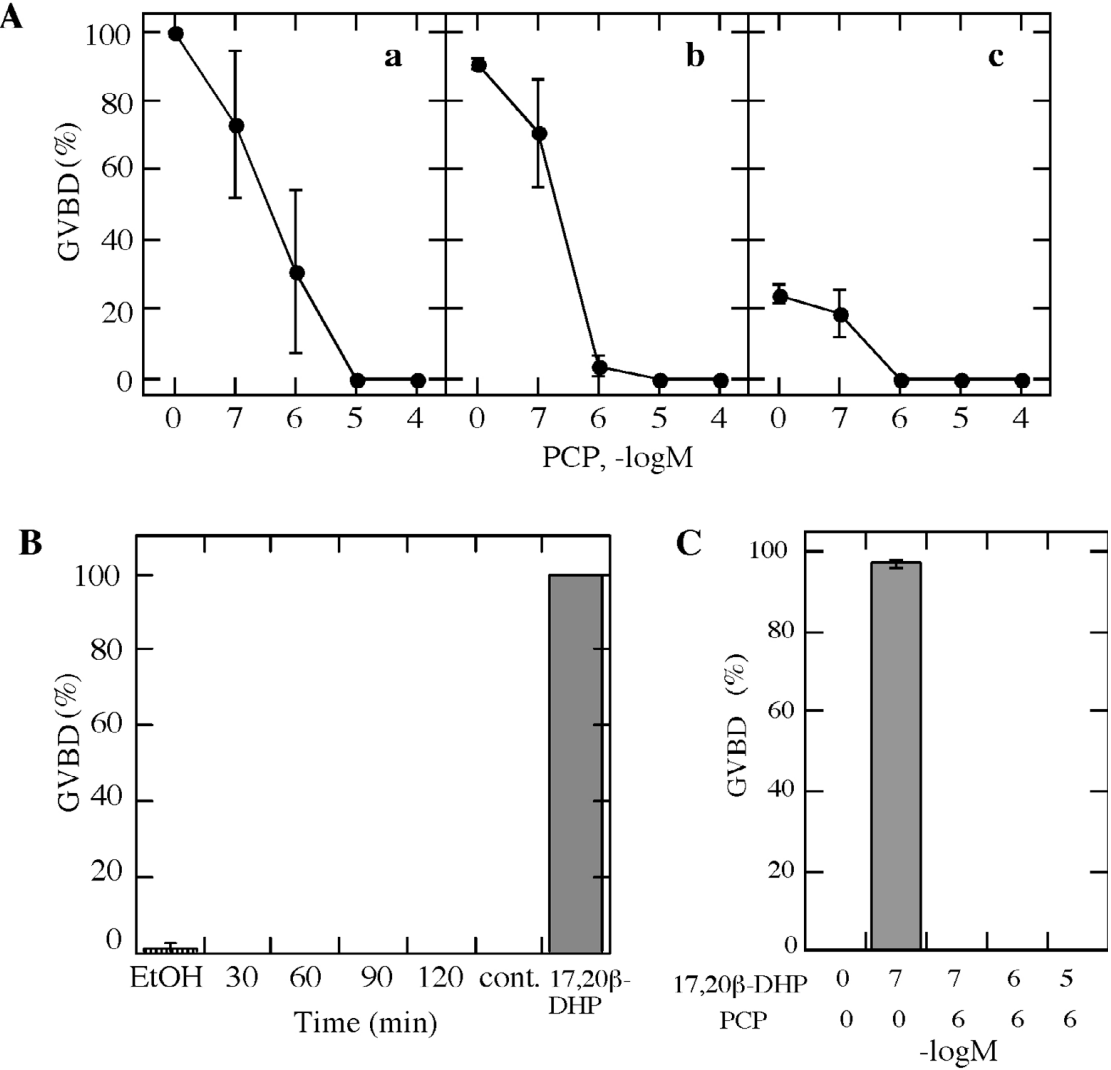
**Inhibition of maturation by PCP**. (A) PCP was added to indicate concentrations, and then maturation was induced by 0.1 μM of 17,20β-DHP (a), 1 μM of DES (b) or 10 μM of TAM (c). (B) Oocytes were treated with 1 μM of PCP for the periods indicated, and then PCP was washed-out by changing the medium three times. Then, maturation was induced by adding 0.1 μM of 17,20β-DHP or ethanol (EtOH). Incubation with the mixture of PCP and 17,20β-DHP is indicated as cont. (C) Oocytes were treated with combinations of indicated concentrations of PCP and 17,20β-DHP. %GVBD was calculated by determining the percentage of oocytes that had undergone germinal vesicle breakdown (GVBD) among more than 20 oocytes cultured for three hours in each experiment. Each value is the mean of three separate experiments using ovaries of three different females. Vertical lines indicate standard deviation.

PCP inhibited oocyte maturation induced by DES (Fig. 4A–b) and TAM (Fig. 4A–c) as well as that induced by 17,20β-DHP (Fig. 4A–a). These results suggested that these compounds induce oocyte maturation by the same mechanism through mPR. An inhibitory effect of PCP on 17,20β-DHP-induced oocyte maturation was observed even at lower concentrations (Fig. 4A–a). IC_50_ for 0.1 μM 17,20β-DHP-induced maturation was estimated as 0.8 μM. When oocytes were pre-incubated with PCP, maturation was completely prevented and the effect was not reduced by washout (Fig. 4B). The result indicates that PCP binds rigidly to the target molecule. Large amounts of 17,20β-DHP could not overcome the inhibitory effect of PCP (Fig. 4C). This result demonstrated that PCP interacts with the target molecules of oocytes to inhibit oocyte maturation but not with steroid, 17,20β-DHP.

### Effect of natural steroids on inducing oocyte maturation

**Figure 5:**
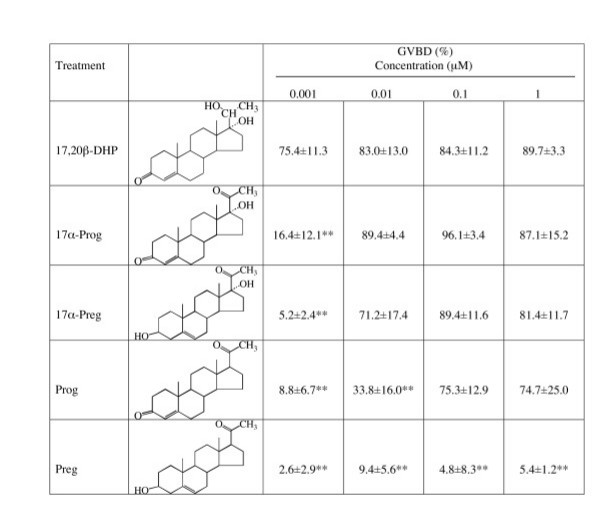
**In vitro induction of germinal vesicle breakdown by natural steroids in zebrafish oocytes**. %GVBD was calculated by determining the percentage of oocytes that had undergone GVBD in a group composed of more than 20 oocytes cultured in parallel for three hours. Each value in the mean (± S.D.) of three separate experiments using ovaries of three separate females. ** indicate significant differences between the %GVBD induced by same concentration of 17,20β-DHP at the P < 0.01 level.

The relative effectiveness of steroids in inducing GVBD was determined (Fig. 5). We selected steroids in which one can consider the effect of alterations of the hydroxyside at C20, C17 and C3. From the difference in potency between 17,20β-DHP and 17α-Prog, it could be concluded that conversion of the 20-keto group to a 20β-hydroxy group was the single stimulatory alteration. Also, a difference in the potency between 17α-steroids and Prog or Preg, showed that addition of a single hydroxyl group at the 17α position increased the ability to induce GVBD. In zebrafish, pregnenolone was almost completely ineffective in inducing GVBD. The result suggests that a change to the 3-keto group is also important for interaction between steroids and the MIH receptor.

## Discussion

In this study, the effects of EDCs on the maturation of zebrafish oocytes were examined. We found that treatment of oocytes with nonsteroidal substances; TAM and 4-OHT, alone induced maturation as did DES. The morphology and an intracellular molecular event induced by EDCs and 17, 20β-DHP were indistinguishable and suggest that, at least qualitatively, EDCs and 17, 20β-DHP induced the same type of maturation.

TAM and its metabolite 4-OHT share structural similarity with DES and have been shown to have estrogenic effects the same as DES [17]. TAM is a non-steroidal anti-estrogen commonly used in the treatment of advanced breast cancer and as adjuvant therapy following surgery in early stage disease [18]. Its use has also been approved for reducing the incidence of breast cancer among high-risk women [19]. TAM acts as an estrogen antagonist in mammary tissue, but mimics the agnostic effects of estrogen in bone and in the cardiovascular system [20]. In the uterus, TAM acts as a partial estrogen agonist [21]. Previously, we predicted that TAM and 4-OHT might have maturation-inducing activity given their structural similarity to DES. As predicted, these compounds did possess maturation-inducing activity. In general, the potency and biological properties of a compound can be predicted from its structure. It is, therefore, possible to clarify the contribution of a particular structure to the biological response of a target. DES and tamoxifens both have an ethylated stilbene. Studies using DES analogues and tamoxifens indicated that an ethylated stilbene is essential to act as a ligand for mPR to induce oocyte maturation. To consider the possible site of interaction between DES and the MIH receptor, we compared the ability of progestines to induce the maturation of zebrafish in this study.

All of the progestins examined except pregnenolone induced a dose-related stimulation of oocyte maturation similar to that induced by 17,20β-DHP, although potencies were significantly different: 17,20β-DHP > 17α-Prog > 17α-Preg> Prog > Preg (Fig. 5). Conversion of the 20-keto group to a 20β-hydroxy group was the single stimulatory alteration to form a natural MIH, 17,20β-DHP. The results show that addition of a single hydroxyl group to position 17α of progesterone also increased the ability to induce GVBD. Conversion of the 3-hydroxy group to a 3-keto group was also had a stimulatory effect because the potency differed between progesterone and pregnenolone and between 17α-Prog and 17α-Preg. This analytical approach to the structure-activity relationships of steroids revealed that 20β-OH, 17α-OH and 3 = O are critical groups that contribute to the interaction with the MIH receptor. The oxygen atom is thought to make a hydrogen bond with residues in the receptor protein. In the case of 17,20β-DHP, it is thought that three oxygen atoms contribute to a hydrogen bond. DES may mimic the hydrogen bond generated by 20β-OH and 3 = O. Loss of a third hydrogen bond may explain the weak activity of DES to induce oocyte maturation.

There have been reported antagonisms and an agonism of MIH-induced oocyte maturation (summarized in Table 1). Pickford and Morris evaluated the effect of methoxychlor on progesterone-induced maturation of *Xenopus laevis *[22]. They showed prolonged pre-treatment of the progesterone pesticide methoxychlor significantly reduced the rate of maturation. Fort et al. also demonstrated the inhibitory effects of EDCs, such as ethinylestradiol, on *Xenopus *oocyte maturation in a series of their study [23]. In fish, Kepon and o,p-DDD reported to antagonize MIH-induced meiotic maturation [24]. In the present study, we have also shown that PCP has a potent inhibitory effect on oocyte maturation in fish. Methoxychlor was also prevented fish oocyte maturation when pre-treated. PCP is a widely used biocide that has been employed as a wood preservative, herbicide and defoliant [25]. Its extensive use and persistence have resulted in significant environmental contamination and potential exposure of the general population. The carcinogenic effects of PCP have been evaluated in several animal bioassays [26]. PCP has been found at 313 of the 1,585 sites in the United States (EPA, 1987). PCP levels in fish used as a biomarker of contamination [27]. This chemical also found in relatively higher levels in wild fish (10 μg/kg) in Japan (Japan Environmental Agency, 1999). The concentrations of PCP to affect zebrafish oocyte maturation are considerably higher than those present even in highly polluted areas of the industrialized world. Then the possibility that PCP affect the oocyte maturation in wild life as an environmental toxicant might be very low. Although the potential to produce adverse reproductive effect is considerable. In fact, the relationship between PCP levels in women with reproductive and other endocrine problems were reported [28,29]. We could not rule out the possibility that PCP concentration *in vivo *may reach to high by biological concentrations and affect the reproductive systems.

**Table 1:** Effects of endocrine-disrupting chemicals on oocyte maturation

compound	Effect on oocyte maturation	Reference
Methoxychlor	inhibition, *Xenopus*	Pickford and Morris [22]
	inhibition, zebrafish	This article
Kepon, o,p'-DDD	inhibition, spotted sea trout	Das and Thomas [24]
Ethinylestradiol	inhibition, *Xenopus*	Fort et al. [23]
DES	stimulation, goldfish zebrafish	Tokumoto et al. [12]
TAM, 4-OHT	stimulation, zebrafish	This article
PCP	inhibition, zebrafish	This article

Although, we have no data to account for the molecular mechanism for preventing maturation by PCP at present, it is hypothesized that PCP acts as an antagonist for 17,20β-DHP, based on the result that no pre-incubation was required for inhibition of 17,20β-DHP-induced oocyte maturation. Previously, we have shown induction of oocyte maturation in fish by an endocrine disrupting chemical (EDC), DES [12]. Recently we have cloned and identified membrane progestin receptor α (mPRα), a strong candidate for MIH receptor [7]. By using the antibody for mPRα, it is suggested that DES induced maturation via interactions with mPRα [12]. Interactions between the MIH receptor and EDCs have been demonstrated previously in fish. Among EDCs, Kepone and o,p'-DDD, have been reported to antagonize binding of 20β-S to the MIH receptor in spotted sea trout, further suggesting that EDCs may interact with mPRs [24]. Direct evidence of the interaction of EDCs with the MIH receptor remains to be obtained.

## Conclusion

Based on our findings that TAM and 4-OHT possess the inducing activity for oocyte maturation as well as DES, ethylated stillbene could be a structure interacting with MIH receptor. These agents induce physiological oocyte maturation. PCP, a widely used biocide, completely blocked oocyte maturation. These results demonstrated that EDCs also disrupt progesterone-dependent system as well as estrogen-dependent one.

The physiological significance of effects of EDCs will be confirmed by binding assay which show direct evidence of the interaction of EDCs with the MIH receptor.

## Authors' contributions

TT and MT carried out assay for oocyte maturation and also participated in the design of the study and drafted the manuscript. YN participated in coordination of the study.
